# Effect of Dexmedetomidine on Preventing Postoperative Agitation in Children: A Meta-Analysis

**DOI:** 10.1371/journal.pone.0128450

**Published:** 2015-05-21

**Authors:** Juan Ni, Jiafu Wei, Yusheng Yao, Xiaoqin Jiang, Linli Luo, Dong Luo

**Affiliations:** 1 Department of Anaesthesiology, West China Second University Hospital, Key Laboratory of Birth Defects and Related Diseases of Women and Children, Sichuan University, Chengdu, Sichuan, China; 2 Department of Cardiology, West China Hospital, Sichuan University, Chengdu, Sichuan, China; 3 Department of Anesthesiology, Fujian Provincial Hospital, Fuzhou, China; Scientific Inst. S. Raffaele Hosp., ITALY

## Abstract

**Background:**

Emergence agitation (EA) is one of the most common postoperative complications in children. The purpose of this meta-analysis is to assess the effect of dexmedetomidine for preventing postoperative agitation in children.

**Methods:**

We searched the Cochrane Central Register of Controlled Trails, MEDLINE, and EMBASE. Randomized controlled trials were included. The following outcome measures were evaluated: incidence of EA, number of patients requiring rescue, time to eye-open, time to extubation, time to discharge from the postanesthesia care unit (PACU).

**Results:**

We analyzed 19 trials (1608 patients) that met the inclusion criteria. Compared with placebo, intravenous dexmedetomidine significantly reduced the incidence of EA [risk ratio (RR) 0.34, 95% confidence interval (CI) 0.25–0.44, *P*<0.00001). Dexmedetomidine also decreased the incidence of severe pain (RR 0.41, 95% CI 0.27–0.62, *P*<0.0001) and requirement of a rescue drug (RR 0.31, 95% CI 0.18–0.53, *P*<0.0001). However, compared with placebo, dexmedetomidine increased the time to eye-open by 0.98 min (*P* = 0.01) and the time to PACU discharge by 4.63 min (*P* = 0.02). Dexmedetomidine was also compared with midazolam, propofol, ketamine, and fentanyl, among others. No significant difference was found in the incidence of EA for most of these comparisons, with the exception of fentanyl and propofol, where dexmedetomidine was more beneficial.

**Conclusions:**

Dexmedetomidine was proved effective for preventing EA and for reducing severe pain and the requirement of rescue drugs. It slightly increased the time to eye-open and the time to PACU discharge. Dexmedetomidine was also more beneficial than propofol or fentanyl in preventing EA.

## Introduction

Emergence agitation (EA) is a state of nonpurposeful restlessness, noncooperation, and inconsolability. It is often accompanied by crying, screaming, thrashing, and disorientation. EA is one of the most common postoperative complications in children, with reported incidences in that population ranging from 10% to 80% [1–3]. The definitive cause of EA is still unclear, but several factors have been implicated, including pain, preoperative anxiety, type of surgical procedure and anesthetic, and personal characteristics of the patient [4]. EA may result in injury to the child, interfering with recovery from surgery and prolonging the stay in the postanesthesia care unit (PACU). Over several decades, numerous interventions have been studied to prevent EA, including the use of opioids, propofol, midazolam, and dexmedetomidine, among others. Dexmedetomidine is a highly selective and specific α2-adrenergic agonist with sedative, analgesic, and anxiolytic effects [5]. Compared with opioids, dexmedetomidine may be more appropriate for children postoperatively because it causes little respiratory depression. Many trials have reported on dexmedetomidine for EA prevention, including those that focused on different dosages, different administration routines, and comparisons with other interventions. The purpose of the article was to review systematically the effects of dexmedetomidine on preventing EA.

## Methods

This meta-analysis was conducted and reported according to the Preferred Reporting Items for Systematic Reviews and Meta-Analysis (PRISMA) (S1 PRISMA Checklist).

### Search strategy

We searched the Cochrane Central Register of Controlled Trails (November 2013), MEDLINE (2003 to November 2013), and EMBASE (2003–2013, week 48). We identified additional studies by reviewing the reference lists of studies and reviews. The following search-term strategy was used: 1) agitation; 2) delirium; 3) excitement; 4) anesthesia; 5) anesthesia; 6) postoperative; 7) operation; 8) surgery; 9) surgical; 10) dexmedetomidine; 11) children; 12) 1 or 2 or 3; 13) 4 or 5 or 6 or 7 or 8 or 9; 14) 10 and 11 and 12 and 13.

### Criteria for study consideration

We included only prospective randomized controlled trials. The participants of the included studies were children between 0 and 18 years who underwent general anesthesia and surgery. We reviewed studies assessing the preventive effect of dexmedetomidine, including dexmedetomidine compared with placebo and other drugs (e.g., opioids, propofol, midazolam) or different doses or administration routes of dexmedetomidine.The outcome measures were the (1) incidence of EA, (2) number of patients requiring rescue, (3) time to eye-open, (4) time to extubation, (5) time to discharge from the PACU.

### Data collection and analysis

The eligibility of a trial to be included was assessed by two coauthors independently. As per Wei et al. [6], titles and abstracts of searched studies were screened for further assessment. Full texts were reviewed as any trial that appeared eligible. Disagreements were resolved by discussion with other authors of our group to achieve consensus.

Data extraction was conducted independently by two of the coauthors using a piloted data extraction form. The following study characteristics were collected: primary author, publication year, country of origin, types of surgery, design (randomized, blind), participant characteristics (age, sample size), intervention (type, dosage, administration route), outcomes of and criteria for EA. For dichotomous data, we recorded the number of participants experiencing the event in each group. Continuous data were extracted using the mean and standard deviation (SD).

Data analysis was performed using Review Manager 5.2 (Cochrane, London, UK). Studies with similar interventions were included in a meta-analysis. Results from dichotomous data were expressed as the risk ratio (RR). Results from continuous data were expressed as the mean difference. According to the heterogeneity of the studies, a fixed-effect model or random-effect model was chosen. Heterogeneity testing was performed with the Z score and χ^2^ statistical analysis, with *p*<0.1 considered to indicate heterogeneity. The results were analyzed using the fixed-effect model if heterogeneity did not exist. When heterogeneity existed, results were analyzed based on the random-effects model. Sensitivity and subgroup analyses were performed to identify the possible sources of heterogeneity. Publication bias was evaluated by Egger’s test using Stata 13.1 software (Stata, College Station, TX, USA). Significance was set at *P*<0.05.

## Results

### Search results and description of the included studies

**Fig 1:**
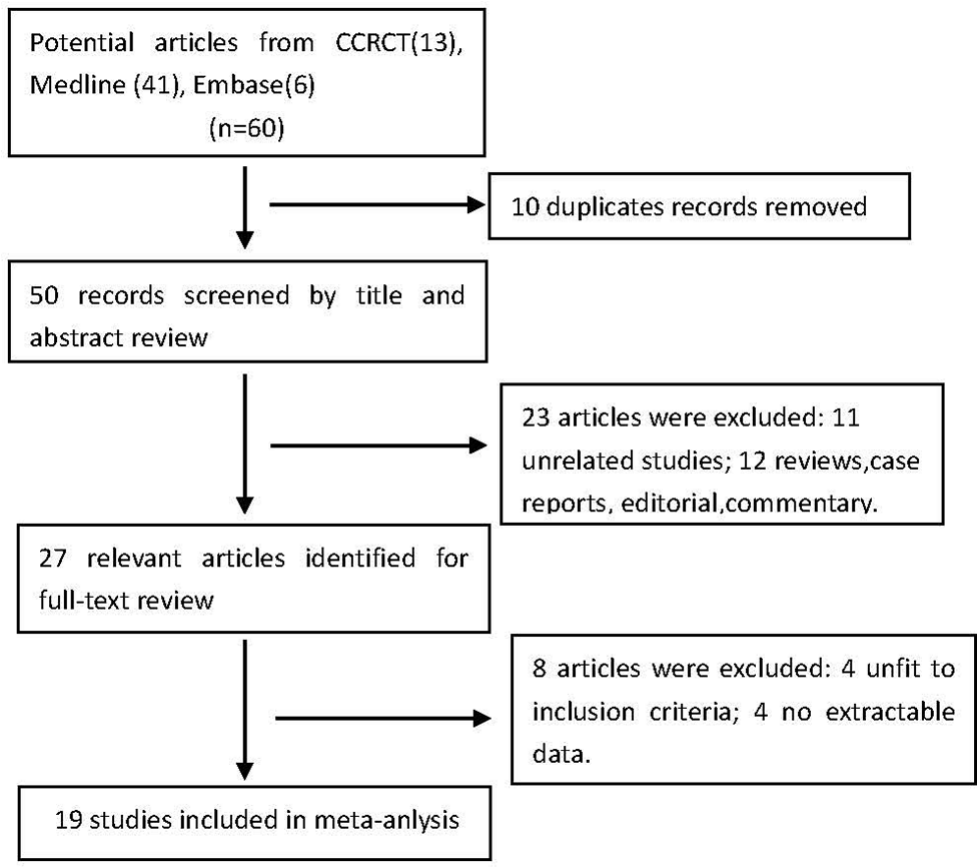
Flow diagram of included/excluded studies.

Fig 1. shows a flow diagram of the trial selection process. A total of 60 potential articles from electronic databases were identified. We reviewed 27 records in full after screening the titles and abstracts. Finally, 19 trials (S1 References) with a total of 1608 patients were included, and relevant data were extracted (Table 1). All of the included studies were of parallel, double-blind, randomized, controlled design. A total of 15 participants in two trials were dropouts [7,8].

**Table 1:** Characteristics of included studies.

Study ID	Age	Surgery	Pts	Anesthesia methods	Groups(n)	Outcome measures
Shukry 2005^[^ ^7^ ^]^	1 to 10 years	Outpatient surgery	50	Sevoflurane, LMA or Intubation	D(23): DEX 0.2ug/kg/h infusion. P(23): NS infusion	Incidence of EA, Time to extubation, Time to discharge from PACU
Patel 2010^[^ ^8^ ^]^	2 to 10 years	Tonsillectomy	133	Sevoflurane and N_2_O, Intubation	D(61): DEX 2ug/kg iv, then 0.7ug/kg/h. F(61): Fentanyl 1ug/kg iv	Incidence of EA, requiring rescue, Time to eye open, Time to extubation
ISIK 2006^[^ ^9^ ^]^	18months to 10 years	MRI scanning	42	Sevoflurane and N_2_O, LMA	D(21): DEX 1ug/kg iv. P(21): NS iv	Incidence of EA, LMA removal time, Time to eye open, Time to discharge from PACU
Koruk 2010^[^ ^10^ ^]^	11.3±7.9 years	Transcatheter atrial septal closure operation	18	Propofol, Intubation	D(9): DEX 1ug/kg iv, then 0.5ug/kg/h. K(9): Ketamine 1mg/kg iv, then 0.5mg/kg/h	Incidence of EA
Pestieau 2011A^[^ ^11^ ^]^	2 to 12 years	Tonsillectomy	101	Sevoflurane and N2O for induction, desfluran and N2O for maintenance, Intubation	D1(25): DEX 2ug/kg iv;D2(25): DEX 4ug/kg iv;F1(26): Fentanyl 1ug/kg iv;F2(25): Fentanyl 2ug/kg iv	Incidence of EA, Requiring rescue
Meng 2012^[^ ^12^ ^]^	5–13 years	Tonsillectomy	120	Propofol and sufentanil for induction, Sevoflurane and remifentanil for maintenance, Intubation	D1(40): DEX 0.5ug/kg iv, then 0.2ug/kg/h;D2(40): DEX 1ug/kg iv, then 0.4ug/kg/h;P(40): Lactated Ringer's	Incidence of EA, Time to extubation, Time to eye open, Time to discharge from PACU
Akin 2012^[^ ^13^ ^]^	2 to 9 years	Tonsillectomy	90	Sevoflurane and N2O, fentanyl, Intubation	D1(45): DEX 1ug/kg intranasal 45-60min before induction.M(45): Midazolam 0.2mg/kg intranasal 45-60min before induction	Incidence of EA; Severe pain, Time to extubation
Sheta 2013^[^ ^14^ ^]^	3–6 years	Dental rehabilitation	72	Sevoflurane,fentanyl, Intubation	D(36): DEX 1ug/kg,1ml intranasal 45-60min before anesthesia induction; M(36): Midazolam 0.2mg/kg(up to maxium 5mg), 1ml intranasal 45-60min before anesthesia induction	Incidence of EA, Time to emergence, Time to discharge readiness
Talon 2009^[^ ^15^ ^]^	1 to 18 years	Reconstructive surgery	93	Sevoflurane and N2O,remifentanil, LMA or Intubation	D(47): DEX 2ug/kg intranasal; M(46): Midazolam 0.5mg/kg, orally with a maximun dose of 20mg	Incidence of EA, requiring rescue
Saadawy 2009^[^ ^16^ ^]^	1 to 6 years	Unilateral inguinal hernia/ orchidopexy	60	Propofol for induction, sevoflurane and N2O for maitenance, bupivacaine for caudal block, LMA	D(30): DEX 1ug/kg caudal injection; P(30): NS caudal injection	Incidence of EA, Severe pain, Time to eye open
Ozcengiz 2011^[^ ^17^ ^]^	3 to 7 years	Esophageal dilatation procedures	100	Sevoflurane and N2O, Intubation	D(25): DEX 2.5ug/kg orally 40–45 min before induction; M(25): Midazolam 0.5mg/kg orally 40–45 min before induction; ML(25): Melatonin 0.1mg/kg orally 40–45 min before induction; P(25): NS orally 40–45 min before induction	Incidence of EA
Pestieau 2011^[^ ^18^ ^]^	6 month to 6 years	Bilateral myringotomy	101	Seoflurane and N2O, Intubation	D1(23): Dexmedetomidime 1ug/kg 1ml intranasal; D2(28): Dexmedetomidime 2ug/kg 1ml intranasal; F(23): Fentanyl 2ug/kg 1ml intranasal; P(27): NS 1ml intranasal	Incidence of EA, severe pain, Time to eye open
Chen 2013^[^ ^19^ ^]^	2 to 7 years	strabismus surgery	78	Sevoflurane, LMA	D1(27): DEX 1ug/kg iv, then 1ug/kg/h; K(27): Ketamine1mg/kg iv, then 1mg/kg/h; P(24): NS 0.25ml/kg for 1 min,0.25ml/kg/h;	Incidence of EA, LMA removal time, Time to discharge from PACU
Ali 2013^[^ ^20^ ^]^	2 to 6 years	Tonsillectomy	120	Sevoflurane and N2O, Intubation	D(40): DEX 0.3ug/kg iv about 5min before the end of surgery; Pr(40): Propofol 1mg/kg iv about 5min before the end of surgery; P(40):NS iv about 5min before the end of surgery	Incidence of EA, Time to extubation, Time to eye open, Time to discharge from PACU
Erdil 2009^[^ ^21^ ^]^	2 to 7 years	Tonsillectomy with or without myringotomy	90	Sevofluran and N2O, Intubation	D(30): DEX 0.5ug/kg iv; F(30): Fentanyl 2.5ug/kg iv; P(30): NS iv	Incidence of EA, Requiring rescue, Severe pain, Time to eye open, Time to extubation
Guler 2005^[^ ^22^ ^]^	3 to 6 years	Tonsillectomy	60	Seoflurane and N2O, Intubation	D(30): DEX 0.5ug/kg iv; P(30): NS 5ml iv	Incidence of EA, Requiring rescue, Severe pain, Time to eye open, Time to extubation
Ibacache 2004^[^ ^23^ ^]^	1 to 10 years	inguinal hernia repair, orchiopexy, circumcision	90	Seoflurane and N2O and caudal block, LMA	D1 (30): DEX 0.15ug/kg iv; D2(30): DEX 0.3ug/kg iv; P(30): NS 10ml iv	Incidence of EA, Time to eye open, Time to discharge form PACU
Olutoye 2010^[^ ^24^ ^]^	3 to 12 years	Tonsillectomy and Adenoidectomy	109	Sevoflurane and N_2_O, Intubation	D1(26): DEX 0.75ug/kg iv; D2(27): DEX 1ug/kg iv; Mor1(30): Morphine 50ug/kg iv; Mor2(26): Morphine 100ug/kg iv	Incidence of EA, Requiring rescue, Time to discharge from PACU
Sato 2010^[^ ^25^ ^]^	1 to 9 years	same day surgery or overnight stay surgery	81	Sevoflurane, LMA	D(39): DEX 0.3ug/kg iv; P(42): NS 5ml iv	Incidence of EA, Time to discharge form PACU

Abbreviation: EA, emergence agitation; DEX, dexmedetomidine; NS: normal saline; LMA, laryngeal mask airway; PACU, post anesthesia care unit.

In 17 trials participants were children aged ≤13 years, whereas in the other two trials patients were 1–18 years of age. Participants in 17 trials were outpatients or short-time inpatients admitted for such interventions as tonsillectomy, myringotomy, and inguinal hernia repair. Another study included 42 patients undergoing magnetic resonance imaging [9]. The last study enrolled 18 patients with congenital heart disease undergoing pediatric cardiac catheterization [10].

In 16 trials, anesthesia was induced and maintained with sevoflurane with or without N_2_O. In one of the other three trials, propofol was administered for anesthesia induction and maintenance [10]. In another trial, participants were induced with sevoflurane and N_2_O and were maintained with desflurane and N_2_O [11]. In the last trial, anesthesia was induced with propofol and maintained with sevoflurane [12]. During anesthesia induction or maintenance, remifentanil, propofol, or fentanyl was administered additionally in six trials [10,12–16].

In three trials, the study drugs were administered 40–60 min before anesthesia induction [13,14,17]. In five trials, the drugs were given as a bolus and then infused until the end of surgery. In one trial, after induction, participants received continuous infusions of the study drugs without a bolus injection [7]. In seven trials, study drugs were injected intravenously after anesthesia induction. In two trials, study drugs were given intranasally after induction [15,18]. In one trial, dexmedetomidine or saline was administered by caudal injection [16].

Regarding outcome measures, 13 trials employed a 4-point scale to evaluate agitation: 1, awake, calm, cooperative; 2, crying, required consoling; 3, irritable/restless, screaming, inconsolable; 4, combative, disoriented, thrashing. In 10 trials, patients with a score of ≥3 were classified as having EA. In the other three trials, patients with a score of ≥2 were regarded as having EA [11,15,18]. Four studies employed a 5-point scale for evaluation: 1, sleeping; 2, awake and calm; 3, irritable and crying; 4, inconsolable crying; 5, severe restlessness and disorientation. With this scoring system, EA was defined as a score of ≥4, which was identical to a score of ≥3 using the 4-point scale. One study did not describe the method of evaluation [10]. The last trial employed the Pediatric Anesthesia Emergence Delirium Scale for evaluation, with a score of ≥10 classified as EA [19].

The incidence of severe pain was evaluated in five trials. We merged the data for the “time to remove the laryngeal mask airway” with the “time to extubation” data because of their similarity. Time to eye-open and time to extubation were evaluated in nine trials. Time to discharge from the PACU was evaluated in eight trials. All outcome measures referring to time were described using minutes.

### Effects of interventions

#### Dexmedetomidine vs Placebo

**Fig 2:**
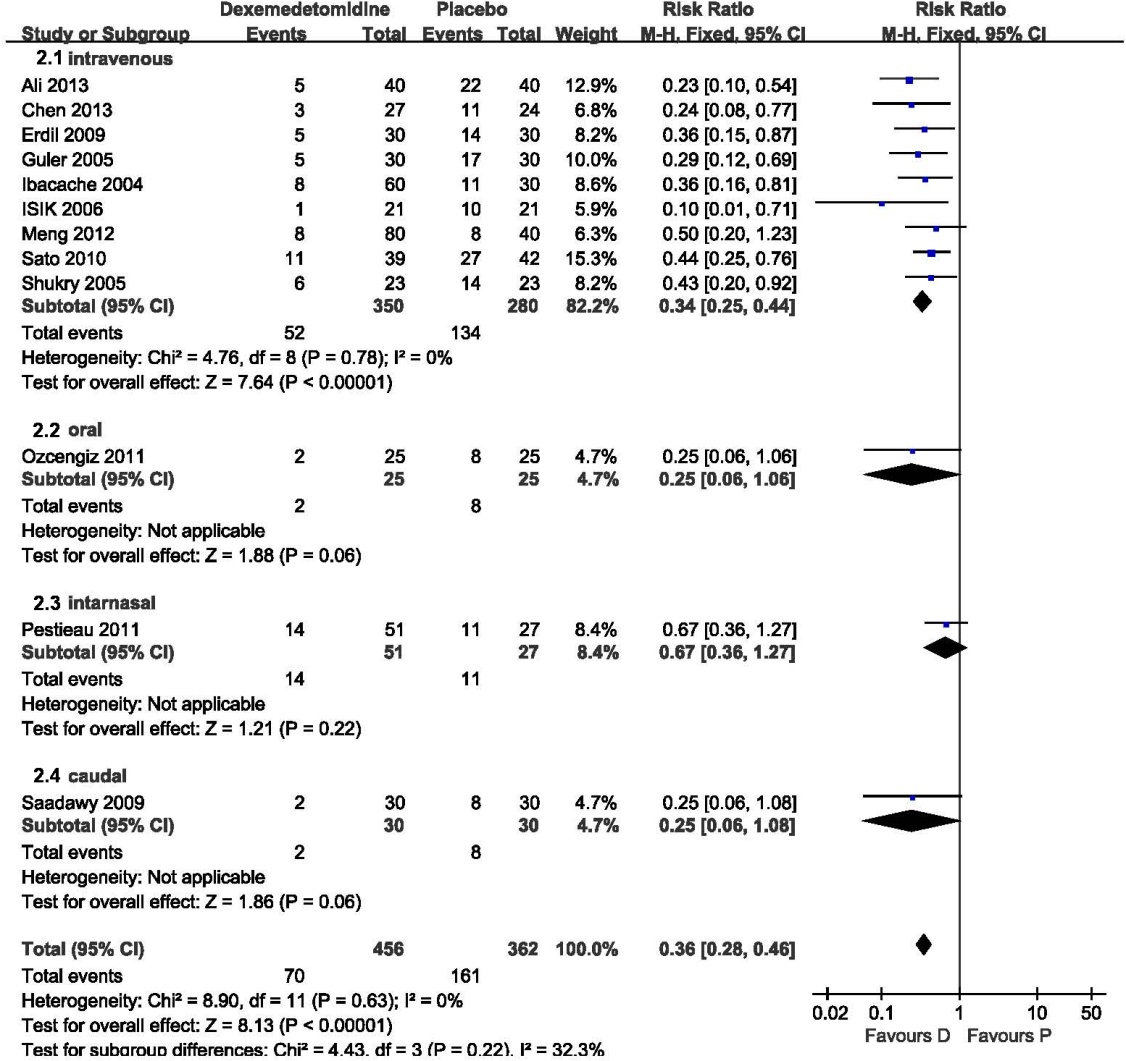
Incidence of emergence agitation (EA): dexmedetomidine vs. placebo. Forest plot shows that the overall effect of pooled trials was in favor of dexmedetomidine. D, dexmedetomidine; P, placebo.

The incidence of EA was compared between dexmedetomidine and placebo in 12 trials (818 patients). Heterogeneity was not observed when these studies were pooled. The pooled analysis showed that dexmedetomidine significantly reduced the risk of EA [RR 0.36, 95% confidence interval (CI) 0.28–0.46; Fig 2]. Subgroup analysis was performed based on the administration route. Intravenous dexmedetomidine significantly decreased the risk of EA (RR 0.34, 95% CI 0.25–0.44; Fig 2). Dexmedetomidine did not reduce the incidence of EA when it was administered via oral, intranasal, and caudal routes. However, there was only one trial for each administration route.

**Fig 3:**
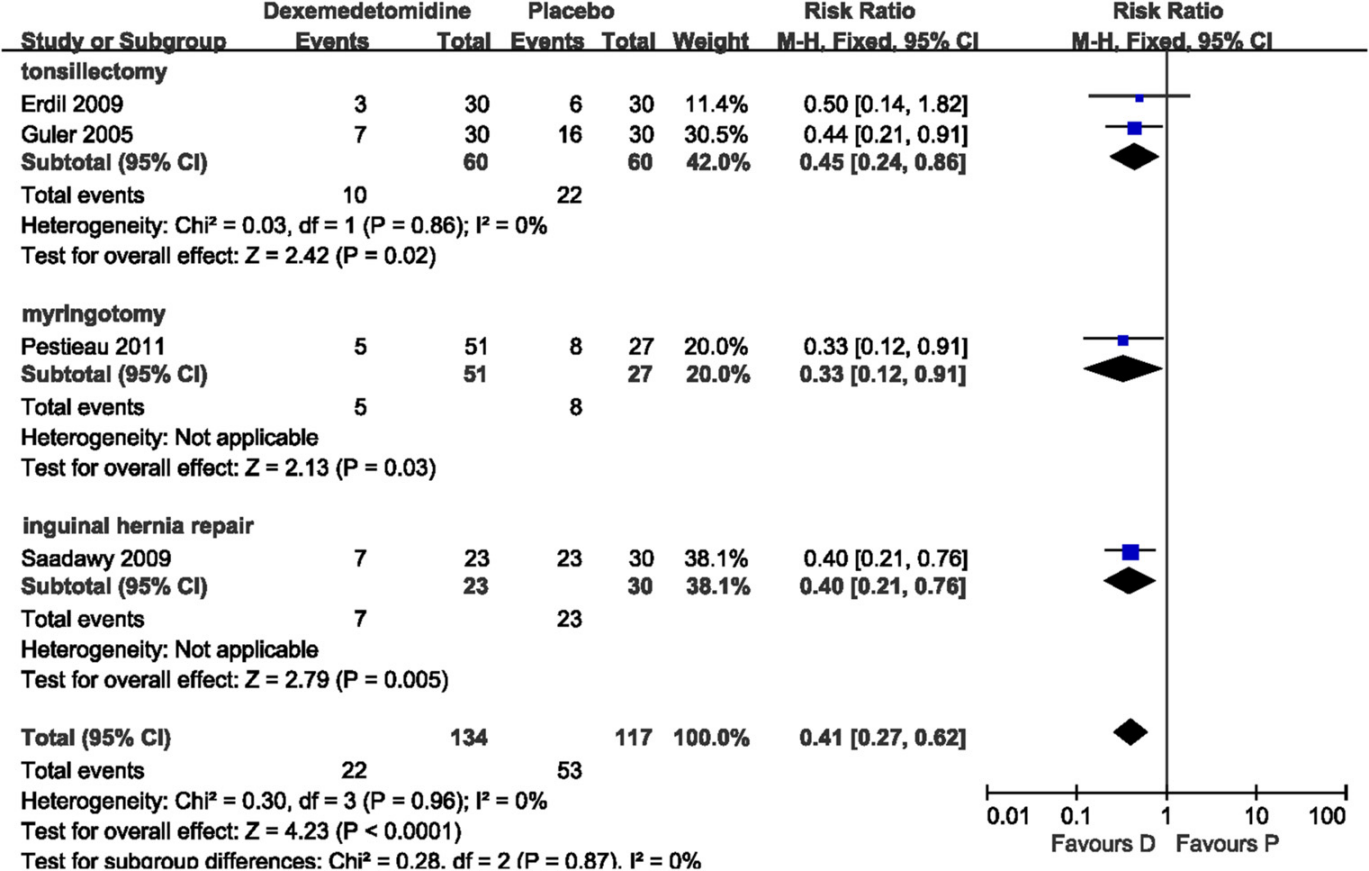
Incidence of severe postoperative pain: dexmedetomidine vs. placebo. Forest plot shows that the overall effect of pooled trials was in favor of dexmedetomidine. D, dexmedetomidine; P, placebo

**Fig 4:**
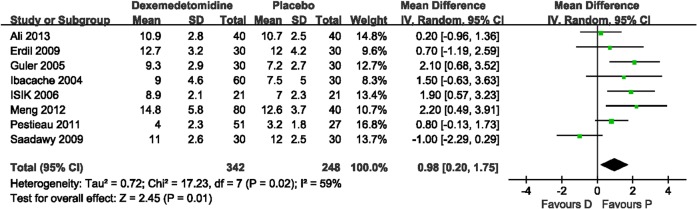
Time to eye-open: dexmedetomidine vs. placebo. Forest plot shows that the overall effect of pooled trials was in favor of placebo. Patients given dexmedetomidine took more time to recover. Heterogeneity was observed when these studies were pooled and the random effects model was chosen for analysis. D, dexmedetomidine; P, placebo

**Fig 5:**
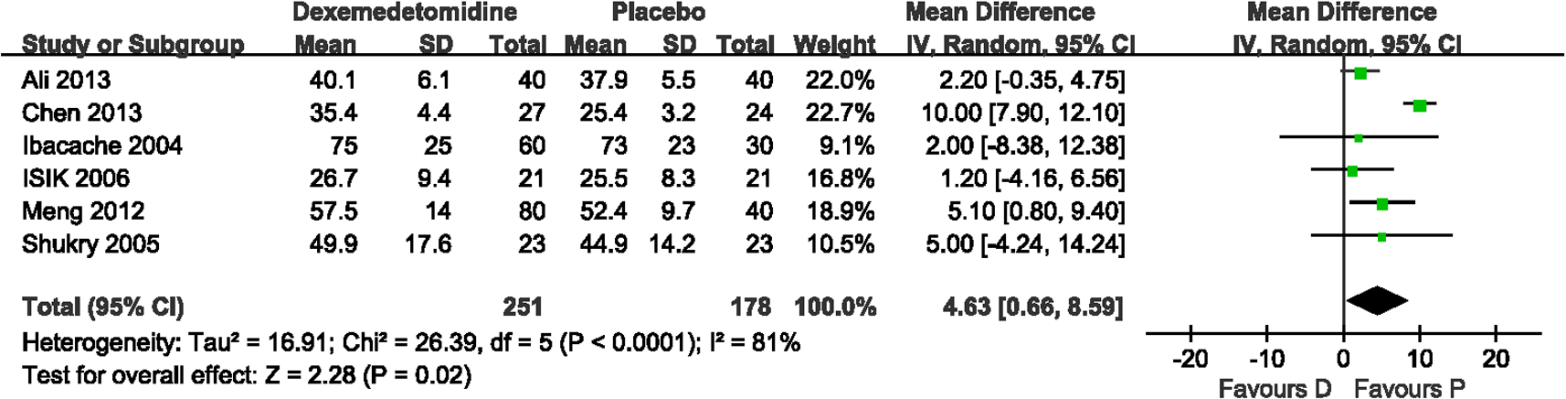
Time to discharge from the postanesthesia care unit (PACU): dexmedetomidine vs. placebo. Forest plot shows that the overall effect of pooled trials was in favor of placebo. Patients given dexmedetomidine stayed longer in the PACU. Heterogeneity was observed when these studies were pooled and the random effects model was chosen for analysis. D, dexmedetomidine; P, placebo

The incidence of severe pain was evaluated in four trials (251 patients). No statistical heterogeneity was found with a pooled analysis. Subgroup analysis was performed based on surgical procedures with different pain intensities. The results showed that dexmedetomidine significantly reduced the risk of severe pain compared with placebo after tonsillectomy (RR 0.45, 95% CI 0.24–0.86; Fig 3). Two studies, Pestieau 2011 and Saadawy 2009, respectively, showed that dexmedetomidine reduced the risk of severe pain after myringotomy (RR 0.33, 95% CI 0.12–0.91; Fig 3) and after inguinal hernia repair surgery (RR 0.40, 95% CI 0.21–0.76; Fig 3) compared with placebo. The number of patients requiring a rescue drug was evaluated in two trials (Erdil 2009, Guler 2005) that included 120 patients. No heterogeneity was observed in the pooled analysis. It showed that dexmedetomidine significantly reduced the requirement of a rescue drug compared with placebo (RR 0.31, 95% CI 0.18–0.53; *P*<0.0001). Time to eye-open was observed in eight studies (590 patients). Heterogeneity was observed when these studies were pooled, which was because of the study of Saadawy 2009. Saadawy 2009 administered the study drugs by caudal injection. The pooled analysis (including Saadawy 2009) showed that dexmedetomidine significantly increased time to eye-open by 0.98 min (mean difference 0.98, 95% CI 0.20–1.75; Fig 4). Seven trials (459 patients) evaluated time to extubation. Heterogeneity was observed when these studies were pooled, which could be because of the study of Guler 2005. No significant difference was shown in the pooled analysis (*P* = 0.05). Time to discharge from the PACU was evaluated in six trials (429 patients). Heterogeneity was observed when these studies were pooled owing to the study of Chen 2013. With the random effects model, dexmedetomidine significantly increased time to discharge from the PACU by 4.63 min (mean difference 4.63, 95% CI 0.66–8.59; Fig 5).

#### Dexmedetomidine vs Midazolam

**Fig 6:**
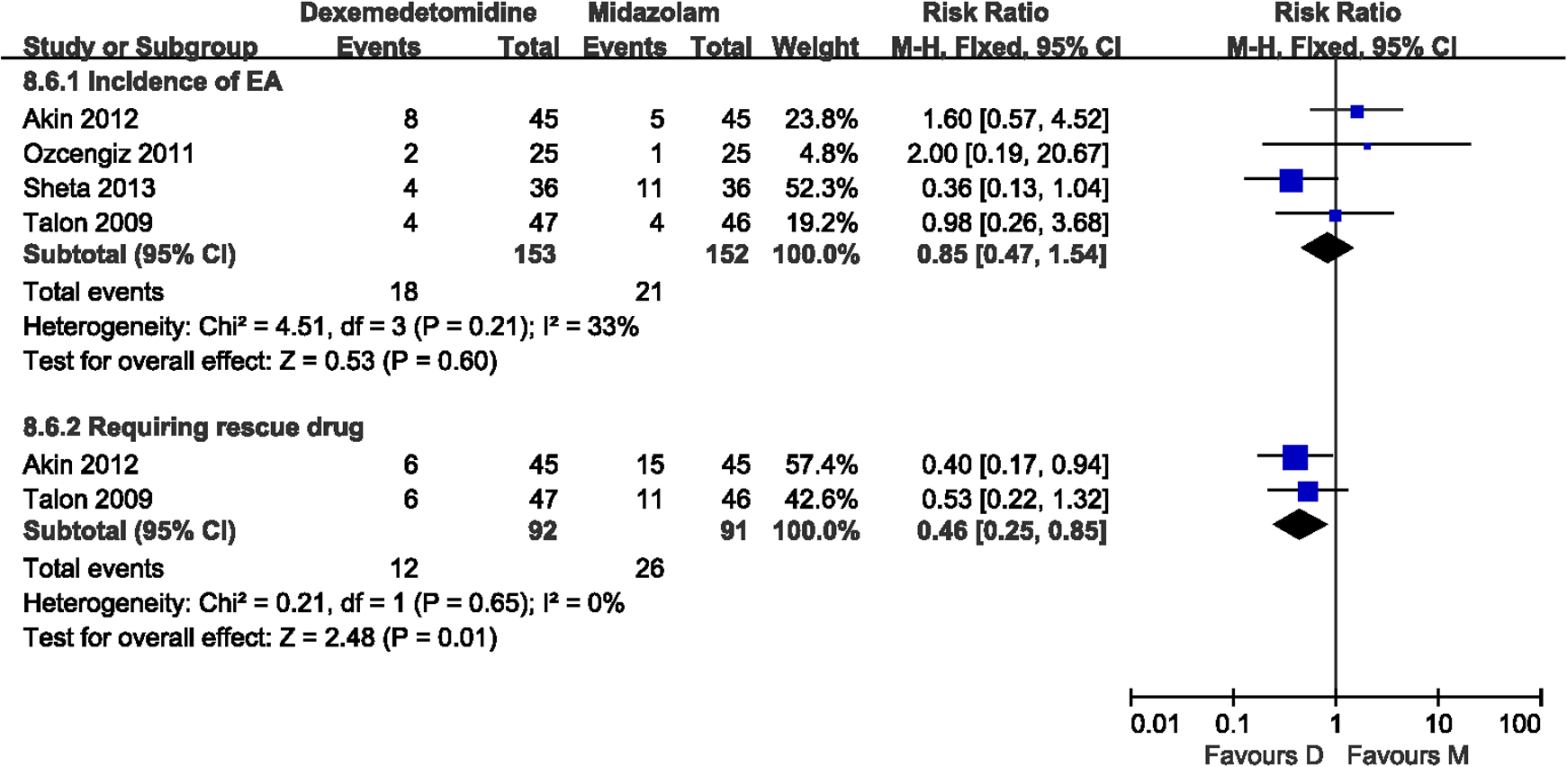
Overall dexmedetomidine vs. midazolam analysis. Incidence of EA was similar for the two groups, with no significant difference. However, the requirment of a rescue drug was less in the dexmedetomidine group than in the midazolam group. D, dexmedetomidine; M, midazolam

Four trials compared the effects of dexmedetomidine and midazolam on preventing agitation. The incidence of EA was evaluated in these four trials. Heterogeneity was not observed when the pooled studies were analyzed. No significant difference (*P* = 0.60) was found between dexmedetomidine and midazolam for reducing the incidence of EA (Fig 6). The number of patients who required a rescue drug was evaluated in two trials. No heterogeneity was observed in the pooled analysis. It showed that dexmedetomidine was more beneficial for reducing the requirement of a rescue drug than midazolam (RR 0.46, 95% CI 0.25–0.85; Fig 6). Only one study (Sheta 2013) evaluated the time to eye-open and time to discharge from the PACU. Neither showed a significant difference. Another study (Akin 2012) evaluated time to extubation. There was no significant difference.

#### Dexmedetomidine vs Propofol

Only one study (Ali 2013) compared the effects of dexmedetomidine and propofol on preventing postoperative agitation. The study evaluated the incidence of EA, time to eye-open, time to extubation, and time to PACU discharge. The result showed that dexmedetomidine was more beneficial than propofol in all outcome measures except time to PACU discharge.

#### Dexmedetomidine vs Ketamine

Two trials (Chen 2013, Koruk 2010) compared the effects of dexmedetomidine and ketamine on preventing postoperative agitation. The incidence of EA was evaluated in both trials. Time to extubation and time to PACU discharge were evaluated in the study by Chen 2013. No significant differences were observed in any of the outcome measures.

#### Dexmedetomidine vs Fentanyl

**Fig 7:**
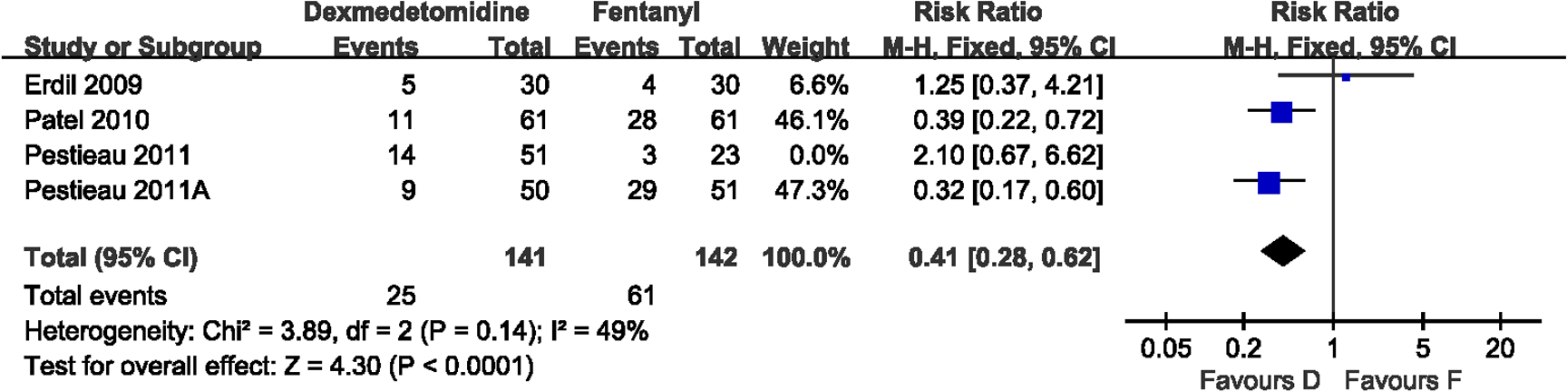
Incidence of EA: dexmedetomidine vs. fentanyl. Forest plot shows that the overall effect of pooled trials without the Pestieau 2011 was in favor of dexmedetomidine. The Pestieau 2011 study was excluded because of clinical and statistical heterogeneity. D, dexmedetomidine; F, fentanyl

Dexmedetomidine and fentanyl were compared in four studies (357 patients). The incidence of EA was evaluated in these four trials. Heterogeneity was observed when these studies were pooled owing to one study (Pestieau 2011). The pooled analysis with a random effects model showed no significant differences between dexmedetomidine and fentanyl. However, the pooled analysis without Pestieau 2011 showed a significant difference in favor of dexmedetomidine(RR 0.41, 95% CI 0.28–0.62; Fig 7). Two trials (Erdil 2009, Pestieau 2011) evaluated the incidence of severe pain, and no significant difference was found. Three trials (Erdil 2009, Patel 2010, Pestieau 2011A) evaluated the incidence of requiring rescue drugs. When these studies were pooled, heterogeneity was observed owing to one study (Patel 2010). The pooled analysis with the random effects model showed no significant difference between dexmedetomidine and fentanyl. Two trials (Patel 2010, Pestieau 2011) evaluated time to eye-open. The pooled analysis of these two trials showed no significant difference. Two trials (Erdil 2009, Patel 2010) evaluated time to extubation. The pooled analysis of these two trials showed a significant difference in favor of dexmedetomidine (mean difference −2.19, 95% CI −3.38 to −0.99).

#### Other comparisons

One study (Olutoye 2010) compared dexmedetomidine with morphine in regard to preventing EA. No significant difference was found in the incidence of EA or in the number of patients requiring rescue drugs. One study (Ozcengiz 2011) compared dexmedetomidine with melatonin, with no significant difference shown in the incidence of EA.

### Adverse effects

Except for one study (Chen 2013), all trials reported the effects of interventions on heart rate and blood pressure. Six studies [8,10,15,18,22,25] reported that the heart rate was lower in the dexmedetomidine group than in the control group. Three studies [8,18,22] showed that the blood pressure was lower in the dexmedetomidine group than in the control group. Bradycardia was recorded in one child given dexmedetomidine (Saadawy 2009).

### Publication bias

Egger’s tests showed that there might be a publication bias for the primary outcome (*P* = 0.045). Regarding the effect of the missing trials, a trim-and-fill analysis was conducted and showed “no trimming performed.”

## Discussion

EA is one of the most common complications of pediatric anesthesia. To date, there is no well-established prophylaxis, although many preventive measures have been studied.

Dexmedetomidine was one of the interventions mentioned above because of its sedative, analgesic, and anxiolytic effects that could contribute to avoiding EA. Our meta-analysis showed that dexmedetomidine, administrated intravenously, decreased the incidence of EA (RR 0.34). Oral and caudal administration seems to be effective for preventing EA (RR 0.25), but there was no significant difference compared with placebo, although there was only one trial each regarding oral [17] and caudal [16] administration. Children accept oral administration more easily than an intravenous injection. Oral dexmedetomidine before anesthesia could ameliorate preoperative anxiety and fear, which was one of the causes of EA. Thus, more prospective studies are required to determine the effect of oral dexmedetomidine on preventing postoperative EA. Caudal dexmedetomidine is invasive and is used only for patients with caudal block. The potential neurotoxicity of dexmedetomidine is another problem that deserves attention [26].

Time to extubation was similar in patients with dexmedetomidine and those with placebo. Compared with placebo, dexmedetomidine significantly increased the time to eye-open by 0.98 min. We did not think, however, that the delay was of notable clinical significance for a procedure that often takes more than 10 min. Additionally, dexmedetomidine prolonged the time to PACU discharge by 4.63 min compared with placebo. In five trials, the Aldrete score or the Postanesthetic Discharge Scoring System was selected for setting the criteria for PACU discharge. Recovery of consciousness was evaluated. The inherent sedative effect of dexmedetomidine might account for the delayed discharge from the PACU.

In our analysis, although dexmedetomidine and midazolam were similarly efficacious in regard to preventing EA, dexmedetomidine incurred less requirment for a rescue drug. These two drugs involve different mechanisms of sedative action. Midazolam depresses the central nervous system by enhancing the effect of the neurotransmitter γ-aminobutyric acid (GABA) on GABA_A_ receptors [27]. Dexmedetomidine acts on the endogenous sleep-promoting pathway to induce a more natural sleep-like status [28]. Pain is another risk factor for EA. Dexmedetomidine provides analgesia via receptors in the spinal cord, which may contribute to the decreased requirementfor a rescue drug.

Patients aged 1–18 years were enrolled in two trials [10,15]. As the age range was scattered and heterogeneity arose, we performed sensitivity analyses to test whether the results would qualitatively change if the trials were included.

We noted significant heterogeneity as a result of one study (Pestieau 2011) when the studies were pooled to evaluate the incidence of EA after administration of dexmedetomidine versus fentanyl. The severe heterogeneity seen in this study could be explained by the different administration routes. The study drugs in the Pestieau 2011 study were administered by nose drops after anesthesia induction, whereas in other studies the drugs were given intravenously. Hence, we did not include this study in the meta-analysis, although the results were included in the forest plot. The analytic results suggested that intravenous dexmedetomidine was more effective than intravenous fentanyl for preventing postoperative EA in children.

Only one trial compared dexmedetomidine with morphine and melatonin, respectively. Neither showed a significant difference. Hence, no further analysis could be conducted.

In this review, the main limitation is the small number of trials comparing dexmedetomidine with other interventions (fentanyl, four trials; midazolam, four trials; ketamine, two trials; propofol, one trial; morphine, one trial). The strength of the evidence may be reduced because of the limited number of trials. Furthermore, the results of Egger’s test suggested a potential publication bias in this review. As the sensitivity of Egger’s test is low when the number of included trials is <20, the trim-and-fill method was performed to reduce the influence of the missing studies.

## Conclusion

Dexmedetomidine was proved to have a beneficial effect in children receiving general anesthesia in regard to preventing EA, avoiding severe pain, and reducing the requirment for a rescue drug. However, it slightly extended the time to eye-open and the time to PACU discharge. The effectiveness of dexmedetomidine for preventing EA was similar to that of midazolam and ketamine. It was more effective, however, than fentanyl or propofol.

## Supporting Information

S1 PRISMA Checklist PRISMA Checklist. (DOC)

S1 References References included in the meta-analysis. (DOCX)
