# Supplementary material for: Effect of Dexmedetomidine on Preventing Postoperative Agitation in Children: A Meta-Analysis
Source: PLoS One. 2015 May 21;10(5):e0128450. doi: 10.1371/journal.pone.0128450 (PMC4440759; doi:10.1371/journal.pone.0128450)
Supplement: S1 References — (DOCX) [file pone.0128450.s002.docx]

**References:**

1. Welborn LG, Hannallah RS, Norden JM, Ruttimann UE, Callan CM （1996） Comparison of emergence and recovery characteristics of sevoflurane, desflurane, and halothane in pediatric ambulatory patients. Anesth Analg 83: 917-920.

2. Voepel-Lewis T, Malviya S, Tait AR （2003） A prospective cohort study of emergence agitation in the pediatric postanesthesia care unit. Anesth Analg 96: 1625-1630, table of contents.

3. Cravero J, Surgenor S, Whalen K （2000） Emergence agitation in paediatric patients after sevoflurane anaesthesia and no surgery: a comparison with halothane. Paediatr Anaesth 10: 419-424.

4. Vlajkovic GP, Sindjelic RP （2007）. Emergence delirium in children: many questions, few answers. Anesth Analg. 2007; 104: 84-91.

5. Bhana N, Goa KL, McClellan KJ （2000） Dexmedetomidine. Drugs 59: 263-268; discussion 269-270.

6. Wei J, Wu T, Yang Q, Chen M, Ni J, Huang D （2011） Nitrates for stable angina: a systematic review and meta-analysis of randomized clinical trials. Int J Cardio 146: 4-12.

7. Shukry M, Clyde MC, Kalarickal PL, Ramadhyani U （2005） Does dexmedetomidine prevent emergence delirium in children after sevoflurane-based general anesthesia? Paediatr Anaesth 15: 1098-1104.

8. Patel A, Davidson M, Tran MC, Quraishi H, Schoenberg C, Sant M, et al. （2010）Dexmedetomidine infusion for analgesia and prevention of emergence agitation in children with obstructive sleep apnea syndrome undergoing tonsillectomy and adenoidectomy. Anesth Analg 111: 1004-1010.

9. Isik B, Arslan M, Tunga AD, Kurtipek O （2006） Dexmedetomidine decreases emergence agitation in pediatric patients after sevoflurane anesthesia without surgery. Paediatr Anaesth 16: 748-753.

10. Koruk S, Mizrak A, Kaya UB, Ilhan O, Baspinar O, Oner U （2010） Propofol/dexmedetomidine and propofol/ketamine combinations for anesthesia in pediatric patients undergoing transcatheter atrial septal defect closure: a prospective randomized study. Clin Ther 32: 701-709.

11. Pestieau SR, Quezado ZM, Johnson YJ, Anderson JL, Cheng YI, McCarter RJ, et al. （2011） High-dose dexmedetomidine increases the opioid-free interval and decreases opioid requirement after tonsillectomy in children. Can J Anaesth 58: 540-550.

12. Meng QT, Xia ZY, Luo T, Wu Y, Tang LH, Zhao B, et al. （2012） Dexmedetomidine reduces emergence agitation after tonsillectomy in children by sevoflurane anesthesia: a case-control study. Int J Pediatr Otorhinolaryngol 76: 1036-1041.

13. Akin A, Bayram A, Esmaoglu A, Tosun Z, Aksu R, Altuntas R, et al. （2012） Dexmedetomidine vs midazolam for premedication of pediatric patients undergoing anesthesia. Paediatr Anaesth 22: 871-876.

14. Sheta SA, Al-Sarheed MA, Abdelhalim AA （2014） Intranasal dexmedetomidine vs midazolam for premedication in children undergoing complete dental rehabilitation: a double-blinded randomized controlled trial. Paediatr Anaesth 24: 181-189.

15. Talon MD, Woodson LC, Sherwood ER, Aarsland A, McRae L, Benham T （2009） Intranasal dexmedetomidine premedication is comparable with midazolam in burn children undergoing reconstructive surgery. J Burn Care Res 30: 599-605.

16. Saadawy I, Boker A, Elshahawy MA, Almazrooa A, Melibary S, Abdellatif AA, et al. （2009） Effect of dexmedetomidine on the characteristics of bupivacaine in a caudal block in pediatrics. Acta Anaesthesiol Scand 53: 251-256.

17. Ozcengiz D, Gunes Y, Ozmete O （2011） Oral melatonin, dexmedetomidine, and midazolam for prevention of postoperative agitation in children. J Anesth 25: 184-188.

18. Pestieau SR, Quezado ZM, Johnson YJ, Anderson JL, Cheng YI, McCarter RJ, et al. （2011） The effect of dexmedetomidine during myringotomy and pressure-equalizing tube placement in children. Paediatr Anaesth 21: 1128-1135.

19. Chen JY, Jia JE, Liu TJ, Qin MJ, Li WX （2013） Comparison of the effects of dexmedetomidine, ketamine, and placebo on emergence agitation after strabismus surgery in children. Can J Anaesth 60: 385-392.

20. Ali MA, Abdellatif AA （2013） Prevention of sevoflurane related emergence agitation in children undergoing adenotonsillectomy: A comparison of dexmedetomidine and propofol. Saudi J Anaesth 7: 296-300.

21. Erdil F, Demirbilek S, Begec Z, Ozturk E, Ulger MH, Ersoy MO （2009） The effects of dexmedetomidine and fentanyl on emergence characteristics after adenoidectomy in children. Anaesth Intensive Care 37: 571-576.

22. Guler G, Akin A, Tosun Z, Ors S, Esmaoglu A, Boyaci A （2005） Single-dose dexmedetomidine reduces agitation and provides smooth extubation after pediatric adenotonsillectomy. Paediatr Anaesth 15: 762-766.

23. Ibacache ME, Munoz HR, Brandes V, Morales AL （2004） Single-dose dexmedetomidine reduces agitation after sevoflurane anesthesia in children. Anesth Analg 98: 60-63, table of contents.

24. Olutoye OA, Glover CD, Diefenderfer JW, McGilberry M, Wyatt MM, Larrier DR, et al. （2010） The effect of intraoperative dexmedetomidine on postoperative analgesia and sedation in pediatric patients undergoing tonsillectomy and adenoidectomy. Anesth Analg 111: 490-495.

25. Sato M, Shirakami G, Tazuke-Nishimura M, Matsuura S, Tanimoto K, Fukuda K （2010） Effect of single-dose dexmedetomidine on emergence agitation and recovery profiles after sevoflurane anesthesia in pediatric ambulatory surgery. J Anesth 24: 675-682.

26. Konakci S, Adanir T, Yilmaz G, Rezanko T （2008） The efficacy and neurotoxicity of dexmedetomidine administered via the epidural route. Eur J Anaesthesiol 25: 403-409.

27. Kupietzky A, Houpt MI （1993） Midazolam: a review of its use for conscious sedation of children. Pediatr Dent 15: 237-241.

28. Coursin DB, Coursin DB, Maccioli GA （2001） Dexmedetomidine. Curr Opin Crit Care 7: 221-226.
